# Association of *AFF3* Gene Polymorphism rs10865035 with Rheumatoid Arthritis: A Population-Based Case-Control Study on a Pakistani Cohort

**DOI:** 10.1155/2021/5544198

**Published:** 2021-05-15

**Authors:** Yasir Ali, Suleman Khan, Yangchao Chen, Nadia Farooqi, Zia-Ul Islam, Mehran Akhtar, Aamir, Aisha Aman, Aftab Ali Shah, Muhsin Jamal, Fazal Jalil

**Affiliations:** ^1^Department of Biotechnology, Abdul Wali Khan University Mardan, Mardan, Pakistan; ^2^Lady Ready Hospital, MTI Peshawar, Peshawar, Pakistan; ^3^School of Biomedical Sciences, Chinese University of Hong Kong, Hong Kong, Pakistan; ^4^Department of Biotechnology, COMSATS University Islamabad, Abbottabad Campus, Abbottabad, Pakistan; ^5^Department of Biotechnology, University of Malakand, Chakdara, Pakistan; ^6^Department of Microbiology, Abdul Wali Khan University Mardan, Mardan, Pakistan

## Abstract

Rheumatoid arthritis (RA) is one of the complex diseases with the involvement of the genetic as well as environmental factors in its onset and severity. Different genome-wide association and candidate gene studies have shown the role of several genetic variants in multiple loci/genes with ethnical and geographical variations. This study was designed to detect the association of a single-nucleotide polymorphism (SNP) rs10865035 in the *AFF3* gene with the genetic background of rheumatoid arthritis (RA) in the Pakistani cohort. A total of 703 individuals, including 409 RA patients and 294 healthy controls, were genotyped using TaqMan assay and Tri primer ARMS-PCR (amplification-refractory mutation system-polymerase chain reaction) methods. The association of rs10865035 with the RA was statistically determined using different models. Interestingly, besides the homozygous recessive model (G/G vs. A/G + A/A) (OR = 1.693(1.06–2.648); *P* = 0.025), all other models, which included the codominant (*χ*^2^ = 5.169; *P* = 0.075), homozygous dominant (A/A vs. G/G + A/G) (OR = 0.867 (0.636–1.187); *P* = 0.41), heterozygous (A/G vs. A/A + GG) (OR = 0.491 (0.667–1.215); *P* = 0.49), and additive model (OR = 0.826 (0.665–1.027); *P* = 0.08) showed insignificant distribution of the genotypes among the cases and controls. These findings suggest that the *AFF3* gene (rs10865035) has no significant role in the onset of RA in the Pakistani population.

## 1. Introduction

Rheumatoid arthritis (RA) is a systemic, chronic, and progressive inflammatory disease. This disease mainly affects multiple joints as well as exhibits extra-articular manifestations such as rheumatoid nodules, systemic comorbidities, and pulmonary involvement of vacuities. The therapeutic revolution in the past few decades that includes the advancement of the new therapeutic options, the development of new criteria for classification, the introduction of early therapy, and the application of new effective strategies has transformed the articular and systemic outcomes in RA [1, 2]. The modern genetic technologies combined with the large and well-characterized clinical cohorts have advanced our understanding of the genetics of this disease. Genome-wide association studies (GWAS) have reported more than a hundred loci associated with the RA risk [3–5]. The presence of genetic polymorphisms in HLA (human leukocyte antigen) and non-HLA genes contributes about 60% to the pathogenesis of RA. Out of HLA genes, the *HLA-DRB1* and associated polymorphisms are crucial in genetic susceptibility of RA, whereas other important non-HLA genes are PTPN22, IL23R, TRAF1, CTLA4, IRF5, AFF3, STAT4, CCR6, and PADI4, etc. [6–9].

The *AFF3* (lymphoid nuclear protein related to the AF4 gene) is located at 2q11.2 position and encodes a protein of 1227 amino acids. This gene is highly expressed in the lymphoid tissue and has been suggested to be involved in its development, while a lower level of expression has been reported in other tissues like the brain and lungs [1, 10–12]. This gene forms a nuclear factor that binds to DNA through its transcriptional activation domain. This property of the *AFF3* gene has made it a strong candidate for autoimmunity in the human cell [13–15]. A large number of studies, including the genome-wide association studies and their meta-analysis, conducted in different populations, have investigated the association of *AFF3* rs10865035, rs1160542, rs17023158, and rs1437377 variants in the pathogenesis of RA [13, 16–20].

The *AFF3* gene is not only important in the susceptibility to RA but also is a good candidate in the assessment of the therapeutic response in individuals [12]. The SNPs in any population are helpful in understanding its genetic diversity and to design a population-specific therapy for RA. Previously, European RA risk loci including *AFF3* rs10865035 were replicated in a small sample set of Pakistani individuals [7]. However, in this study, the association of *AFF3* rs10865035 with RA was investigated in another sample set of Pakistani origin and was merged with our previous data for statistical significance.

## 2. Methodology

### 2.1. Study Subjects

A cohort of 703 individuals of Pakistani origin (409 RA cases and 294 healthy controls) were included in this study after getting their informed consent. The study cohort was divided into group A (*n* = 203) and group B (*n* = 500). The dataset of group A was taken from our previous study [7]. The details of group B are given in Table 1. The cases were clinically diagnosed by certified rheumatologists following American College of Rheumatology (ACR) criteria [1], and the data including their clinical and demographic information were recorded on a specially designed questionnaire. A comparison of the RA cases and controls showed that the level of inflammatory markers like rheumatoid factor (RF) and erythrocyte sedimentation rate (ESR) was significantly higher in cases. The participants having no immunological disease/symptoms were counted as controls in this study. The study protocol was approved by the Ethical Review Board of LRH and AWKUM (Abdul Wali khan University Mardan).

### 2.2. DNA Extraction and Genotyping

Whole blood samples were collected and processed for group B (*n* = 500) as previously done by Jalil et al., 2013, for group A (*n* = 203). The genomic DNA was extracted using an organic phenol-chloroform method [21]. The genotyping of the *AFF3* gene variant rs10865035 was carried out using TaqMan genotype SNP assay (Applied Biosystems) and ARMS-PCR techniques. The individuals in group A (*n* = 203) were analyzed through TaqMan assay where the PCR amplification was performed in 384-well plates on dual block GeneAmp PCR system 9700 (Applied Biosystems), following the manufacture's protocol [7]. The remaining 500 samples were analyzed through ARMS-PCR using a set of three primers, including two forward and a common reverse primer (F1 (for A allele): TTTAAAACCTCTATCTGGGGAAAAA, F2 (for G allele): TAAAACCTCTATGGGGAAAAG and R: CCCCTCTAATAGTCAATCAATCAAAATA).

The PCR amplification was performed in 96-well plates on a thermocycler (T100, Bio-Rad). The amplification conditions were set as the initial denaturation at 94°C for 5 minutes, followed by 35 cycles of denaturation at 94°C for 30 seconds, annealing for 30 seconds at 58°C, and extension at 72°C for 1 minute. The final elongation was performed at 72°C for 5 minutes. The PCR products were resolved in 2% agarose gel, and the genotype calls for each subject were recorded using the visual inspection method of the gel.

### 2.3. Statistical Analysis

The association of *AFF3* rs10865035 with RA was tested through different statistical models such as codominant, homozygous dominant, homozygous recessive, heterozygous, and additive. The data were further checked for deviation from the Hardy–Weinberg Equilibrium (HWE), and the error in genotyping was measured by repeating 10% of the samples. The association of *AFF3* rs10865035 with RA was measured by calculating the odds ratio (OR) at 95% confidence interval (CI) using chi-square (*χ*^2^) and Fisher's exact test. The *P* value < 0.05 was considered statistically significant.

## 3. Results

The data of the current study (*n* = 500) were combined with our previous findings (*n* = 203) to make the sample size more effective. In the codominant model, the frequency of the genotypes was found almost similar in both the cases and controls (cases: A/A 133 (32.52%), A/G 210 (51.35%), and G/G 66 (16.14%); controls: A/A 105 (35.72%), A/G 159 (54.09%), and G/G 30 (10.21%), *χ*^2^ = 5.169; *P* = 0.07). Similarly, in the homozygous dominant model, no significant difference was observed in the distribution of A/A vs. G/G + A/G genotypes in the study subjects (OR = 0.867 (0.636–1.187); *P* = 0.41). However, a significant distribution was determined in the homozygous recessive model by comparing G/G vs. A/G + A/A (OR = 1.693 (1.06–2.648); Pe = 0.02). Furthermore, an insignificant distribution of A/G vs. A/A + GG genotypes was observed in the heterozygous model (OR = 0.491 (0.667–1.215); *P* = 0.49) whereas in the additive model, the alleles (A vs. G) were distributed insignificantly among the cases and controls (OR = 0.826 (0.665–1.027); *P* = 0.08). The association results, *P* values along with ORs, are given in Table 2.

## 4. Discussion

Rheumatoid arthritis (RA) is an autoimmune disease that disrupts the normal physical activities of the patients [22, 23]. It causes inflammation in joints which results in damaging the articular cartilage along with the synovial hyperplasia that ultimately leads to consistent pain and permanent disability. In RA, the genetic risk factors (50–60%) are triggered by various environmental factors (40%) [23–25]. Among the genetic factors, the major histocompatibility complex (MHC) and non-MHC loci are accountable for ∼23% of the genetic risk which indicates that there are still undiscovered risk alleles [18]. Furthermore, the association studies have also reported >150 SNPs located at more than 70 gene loci [18, 26–28]. Another gene-based association study that was conducted on European (14,361 cases and 43,923 controls) and Asian populations (4,873 cases and 17,642 controls) identified 221 newly RA-associated genes including 76 European-specific, 74 Asian-specific, and 71 genes which were found overlapped among both the populations [5]. Other studies that followed different study protocols have either validated the role of some of the reported genes or have determined the association of novel genes and polymorphisms in both European and Asian individuals [1, 7, 17, 18, 29, 30].

The aim of this study was to assess the association of the *AFF3* rs10865035 with the genetic background of RA in the Pakistani population. The replication of known genetic polymorphism will enhance our understanding of the ethnogenetic heterogeneity and homogeneity of the Pakistani population with others. The understanding of replication patterns among different populations would help us in the development of a common therapy for disease management.

Previously, we determined an insignificant association (*P* = 0.117) of *AFF3* gene polymorphism rs10865035 in a small sample set (*n* = 213) of Pakistani origin [7]. Therefore, the current study was aimed to replicate these findings in a larger sample (*n* = 500) set of the same ethnicity. To enhance the statistical significance of this study, our previous data of 203 individuals were merged with the current data of 500 subjects. These statistical analyses showed that *AFF3* gene polymorphism rs10865035 has no association with RA in the Pakistani population (Table 2).

The *AFF3* gene encodes a DNA interacting nuclear factor having a transcriptional activation domain that makes it a strong candidate gene in autoimmunity. Previously, more than 50 genetic polymorphisms have been mapped in the same gene [13]. Also, an extended study was conducted on a sample set of 6819 RA patients and 12650 healthy controls, provided a convincing proof for the association of *AFF3* gene variants rs10865035 (OR: 1.12 (1.07–1.17); *P* = 2.8 × 10^−7^) and rs1160542 (OR: 1.12 (1.05–1.20); *P* = 0.001) as novel susceptibility targets [13]. Using these findings, another group of researchers investigated the role of *AFF3* gene polymorphisms (rs10865035 and rs1160542) with respect to anti-TNF treatment in RA (coefficient −0.14 (95% CI −0.25 to −0.03), *P* = 0.01) [31]. Furthermore, a GWAS meta-analysis by Stahl et al. (2010) also confirmed the association of *AFF3* gene polymorphism rs10865035 (OR: 1.12 (1.07–1.17); *P* = 2.0 × 10^−6^) with RA in a sample set of 41,282 individuals (12,307 RA cases and 28,975 controls) of European descent [18].

In order to explore population-specific as well as European-overlap susceptibility loci/genes, a GWAS was carried out in the Korean population. In this study, 1519 RA cases and 1476 healthy controls were genotyped for 441,398 single-nucleotide polymorphisms, and along with many other susceptibility signals, *AFF3* rs10865035 was successfully associated with the RA at genome-wide significance level (*P* < 5 × 10^−08^) [19]. Another replication study, conducted by Prasad et al. [17] on 983 RA cases and 1007 controls of North Indian descent, established the association of seven genes with the RA. They selected 42 candidate genes/loci (3 Asian and 39 European) and tested 603 SNPs, which were either index SNPs or were surrogate SNPs, using Infinium Human 660w-quad microarray methods for genotyping. Out of 12 SNPs, tested in *AFF3* gene, 3 SNPs were found to be significantly associated with RA (rs17023158, OR: 1.45 (1.12–1.88), *P* = 0.005; rs6706188, OR: 0.81 (0.7–0.94), *P* = 0.005; rs1437377, OR: 0.77 (0.65–0.92), *P* = 0.003) [17]. However, rs10865035 was not included in their SNPs array. Similarly, a sequenom mass array-based meta-analysis conducted on European individuals (3311 RA cases and 3709 controls) investigated another SNP rs1160542 and obtained a significant association with RA (OR: 1.08 (1.01–1.16), *P* = 0.029) [20]. In addition, the AFF3 gene has been detected to be significantly associated (OR: 1.25 (1.13–1.39); *P* = 2.05 × 10^−5^) with juvenile idiopathic arthritis (JIA) as well [16]. It was further reported that, besides, its role in RA, the *AFF3* gene shares the same genetic basis with the systemic lupus erythematosus (SLE). A Chinese case-control study (868 SLE patients and 975 controls) observed significant association of the *AFF3* rs10865035 with SLE (OR: 1.26 (1.11–1.44); *P* = 4.81 × 10^−4^) [32]. Furthermore, studies have shown that the *AFF3* gene was initially linked to type 1 diabetes (T1D), and then, later, its role was determined in 16 different autoimmune diseases including RA and SLE [33, 34].

In conclusion, our previous study [7] regarding the association of *AFF3* rs10865035 with RA was further validated using different statistical models in a larger sample set (*n* = 703). This reaffirmation with different statistical tools suggests the nonsignificance of *AFF3* rs10865035 in a Pakistani cohort. However, there is a possibility of the existence of ethnic variability and we urge the need for multiethnic large population-based study in order to understand the exact mechanism of pathogenicity as well as the evolutionary background of the genetic factors involved.

## Figures and Tables

**Table 1:** Characteristic of rheumatoid arthritis patients and controls.

Characteristics	Cases (*n* = 409)	Controls (*n* = 294)
Group A*∗*	159	44
Group B	250	250
Mean age, years ± SD of group A*∗*	39.1 ± 13.0	41.2 ± 12.0
Mean age, years ± SD group B	43.5 ± 14.5	42 ± 12.6
Disease duration in years, mean ± SD	4.1 ± 3.7	NIL
Seropositive antibody, mean ± SD	100% (RF positive)	NIL
ESR, mean ± SD	40.60 (±15.8)	NIL

*∗*Data obtained from our previous study [7].

**Table 2:** Statistical analysis of the *AFF*3 polymorphism (rs340630) in RA cases and controls.

Statistical models	Genotypes	Cases (*n* = 409)	Controls (*n* = 294)	OR 95% CI	*χ* ^2^ value	*P* value
Codominant	A/A	133 (32.52%)	105 (35.72%)	—	5.169	0.07
	A/G	210 (51.35%)	159 (54.09%)			
	G/G	66 (16.14%)	30 1(0.21%)			
Homozygous dominant	A/A	133 (32.5%)	105 (35.7%)	0.867	—	0.41
	A/G + G/G	276 (67.5%)	189 (64.3%)	(0.636–1.187)		
Homozygous recessive	G/G	66 (16.1%)	30 (10.2%)	1.693	—	0.02
	A/A + A/G	343(83.9%)	264 (89.8%)	(1.06–2.648)		
Heterozygous dominant	A/G	210 (51.3%)	159 (54%)	0.491	—	0.49
	A/A + G/G	199 (48.7%)	135 (46%)	(0.667–1.215)		
Additive	A	476 (58.1%)	369(62.3%)	0.826	—	0.08
	G	342 (41.9%)	219 (37.3 %)	(0.665–1.027)		

## Data Availability

All the data used to support the findings of this study are cited in the text.
